# CDK5 functions as a tumor promoter in human colorectal cancer via modulating the ERK5–AP-1 axis

**DOI:** 10.1038/cddis.2016.333

**Published:** 2016-10-13

**Authors:** Kangmin Zhuang, Juchang Zhang, Man Xiong, Xianfei Wang, Xiaobei Luo, Lu Han, Yan Meng, Yali Zhang, Wenting Liao, Side Liu

**Affiliations:** 1 Guangdong Provincial Key Laboratory of Gastroenterology, Department of Gastroenterology, Nanfang Hospital, Southern Medical University, Guangzhou, Guangdong, China; 2 Department of Gastroenterology, Affiliated Hospital of North Sichuan Medical College, Nanchong, Sichuan, China; 3 Department of Pathology, Nanfang Hospital, Southern Medical University, Guangzhou, Guangdong, China; 4 Department of Pathology, School of Basic Medical Sciences, Southern Medical University, Guangzhou, Guangdong, China

## Abstract

Abnormal expression of cyclin-dependent kinase 5 (CDK5) has been found in several human cancers, whereas the role of CDK5 in the malignant development of colorectal cancer (CRC) has not been well characterized. Here we investigated the role of CDK5 in CRC and found that its expression was much higher in CRC tissues than that in normal tissues with a higher expression level of CDK5 closely correlating to advanced American Joint Committee on Cancer (AJCC) stage, poor differentiation, increased tumor size and poor prognosis of CRC. Biological function experiments showed that CDK5 regulated CRC cell proliferation and metastasis ability. Whole-genome microarray analysis, co-immunoprecipitation, *in vitro* kinase assay, western blotting, luciferase reporter assays and electrophoretic mobility shift assay (EMSA) showed that CDK5 could directly phosphorylate ERK5 at threonine (Thr) 732 and finally modulate the oncogenic ERK5–AP-1 axis. Further researches showed that CDK5–ERK5–AP-1 axis could promote progression of CRC carcinogenesis and had a significant correlation in human CRC samples. In summary, this study revealed the functional and mechanistic links between CDK5 and the oncogenic ERK5–AP-1 signaling pathway in the pathogenesis of CRC. These findings suggest that CDK5 has an important role in CRC development and may serve as a potential therapeutic target for CRC.

Colorectal cancer (CRC) is one of the most common malignancies in the world involving progressive disruption of epithelial cell proliferation, apoptosis, differentiation and survival mechanisms.^1, 2^ The CRC carcinogenesis is a multistep and multi-factorial process related to various genetic and epigenetic alterations, including the activation of various oncogenes or inactivation of tumor-suppressor genes.^3, 4^ However, the power of many existing biomarkers in early diagnosis or predicting the clinical outcome of individual tumors is limited owing to the great heterogeneity of this cancer. Thus research of the molecular mechanism that is responsible for the initiation and progression of CRC about the biomarkers could help to identify potential biomarkers, which may facilitate efficient predictive and therapeutic strategies.

Among the cyclin-dependent kinase (CDK) family, CDK5 is an unusual member with specific functions. Though CDK5 is ubiquitously expressed, previous studies about CDK5 were mainly focused on neuronal origin. Unlike other mitotic CDKs, CDK5 is activated by binding to p35 or p39.^5^ In the central nervous system, CDK5 has been proved as a key regulator of neuronal migration, synaptic activity and neuronal cell survival and death.^6, 7, 8^ Over the past decade, an increasing body of evidence has suggested that CDK5 may also have a significant role in the tumorigenesis of multiple organs, such as breast cancer, pancreatic cancer and neuroendocrine thyroid carcinoma.^9, 10, 11^ However, the knowledge on the role and underlying mechanism of CDK5 in CRC remains poorly unknown.

In the present study, we sought to investigate the clinicopathological significance of CDK5 in CRC and its role in CRC development. We found that CDK5 and its activator p35 showed higher expression levels in CRC tissues than paired normal tissues. In addition, high expression level of CDK5 was correlated to the aggressive characteristics (American Joint Committee on Cancer (AJCC), tumor differentiation, tumor size and nodal metastasis) and poor survival of patients. Furthermore, CDK5 might promote proliferation, tumor formation and invasion of CRC partly via modulating the ERK5–AP-1 signaling axis.

## Results

### CDK5 and p35 were both upregulated in CRC

**Figure 1:**
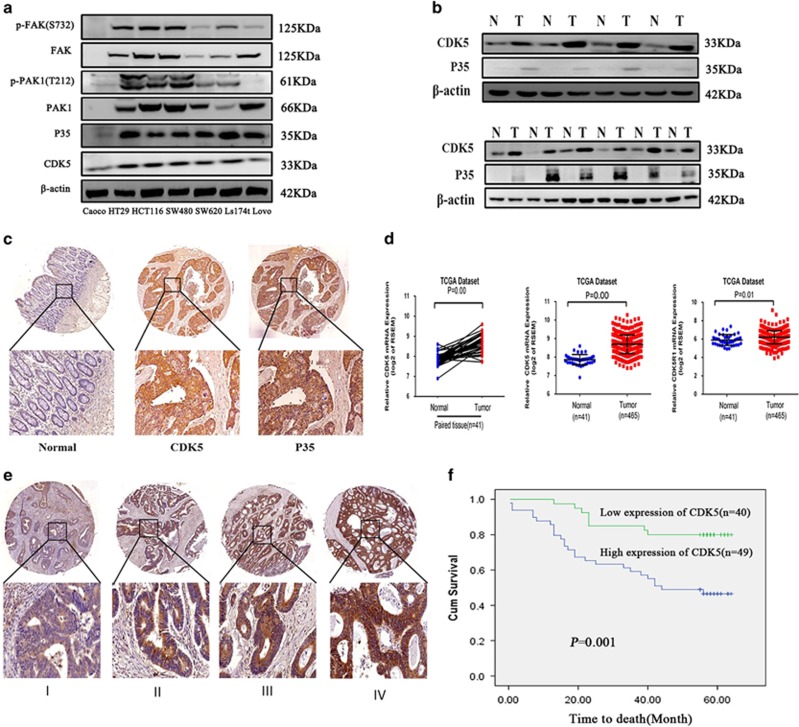
Expression of CDK5 and p35 in CRC tissues and cell lines. (**a**) CDK5, p35 and its kinase activity expression was detected by western blotting in CRC cell lines. (**b**) The protein level of CDK5 and p35 was detected in 10 human CRC tissues (T) and their adjacent normal intestine epithelial tissues (N). (**c**) IHC staining of CDK5 and p35 was performed in the TMA containing 89 colon cancer cases. (**d**) The mRNA level of CDK5 and p35 was shown by a published colorectal cancer data set of TCGA (*n*=465). (**e**) IHC staining of CDK5 expression in human CRC (AJCC stages I–IV). (**f**) Kaplan–Meier overall survival curves for patients with CRC stratified by low (*n*=40) and high (*n*=49) expression of CDK5 (*P*=0.001)

The protein levels of CDK5 and its activator p35 varied in seven CRC cell lines, including Caco-2, HT29, HCT116, SW480, SW620, Ls174t and Lovo. The expression of CDK5 and p35 was detected in the seven CRC cell lines mentioned above. Furthermore, the kinase activity of CDK5 was evaluated by detecting the phosphorylation level of FAK at serine 732 and PAK1 at Thr212, which had already been demonstrated as CDK5's substrates and had been used to evaluate its kinase activity.^10, 12, 13, 14^ Interestingly, CDK5 expression and its kinase activity was relatively higher in aggressive cell lines HCT116 and SW480 than that in less aggressive cell lines Caco-2 and Lovo (Figure 1a). Western blotting and immunohistochemistry (IHC) staining showed that the expression of CDK5 and p35 protein was significantly upregulated in the CRC tissues (T) compared with their adjacent normal intestine epithelial tissues (N) (Figures 1b and c). Furthermore, data obtained from published CRC patient gene expression profiles (The Cancer Genome Atlas (TCGA), *n*=465) revealed a relative higher CDK5 and p35 mRNA expression in CRC tissue compared with normal tissue (Figure 1d).

### CDK5 overexpression was associated with progression and poor survival of CRC

To investigate the clinical significance of upregulation of CDK5 in CRC, CDK5 protein expression was examined in tissue microarray (TMA) containing a total of 89 colon cancer patients using IHC staining (Supplementary Table S1). As a result, CDK5 protein was detected in 85 of the 89 (95.5%) cases of CRC tissue samples with 49 (55.1%) cases displaying high CDK5 expression and 40 (44.9%) cases showing a relatively low CDK5 expression (Table 1). Besides, Pearson's chi-square tests revealed that the expression of CDK5 was significantly correlated with AJCC (*P*=0.01), tumor differentiation (*P*=0.029), tumor size (*P*=0.001) and nodal metastasis (*P*=0.027) (Table 1 and Figure 1e). Notably, Kaplan–Meier survival analysis revealed that patients with high CDK5 expression had poorer overall survival than patients with low CDK5 expression (44 *versus* 54 months, *P*=0.001) (Figure 1f).

Additionally, univariate Cox regression model analysis revealed that poor survival was significantly associated with AJCC (hazard ratio (HR): 3.790, 95% confidence interval (95% CI): 1.889–7.605; *P*<0.001), tumor differentiation (HR: 2.434, 95% CI: 1.345–4.405; *P*=0.003), tumor size (HR: 2.462, 95% CI: 1.147–5.284; *P*=0.021), nodal metastasis (HR: 3.135, 95% CI: 1.588–6.188; *P*=0.001) and CDK5 expression (HR: 3.457, 95% CI: 1.563–7.645; *P*=0.002). Based on the results of the univariate survival analysis, multivariate survival analysis was performed. After adjustment, tumor size, tumor differentiation, AJCC stage and CDK5 expression were identified as covariates. Nodal metastasis was excluded from the multivariated survival analysis owing to its interactions with AJCC stage. The outcome suggested that AJCC stage (HR: 2.603, 95% CI: 1.264–5.358; *P*=0.009) and CDK5 expression (HR: 2.428, 95% CI: 1.089–5.413; *P*=0.030) were both considered as the independent risk predictors for poor overall survival (Supplementary Table S2).

### CDK5 promoted the proliferation ability of CRC cells *in vitro*

**Figure 2:**
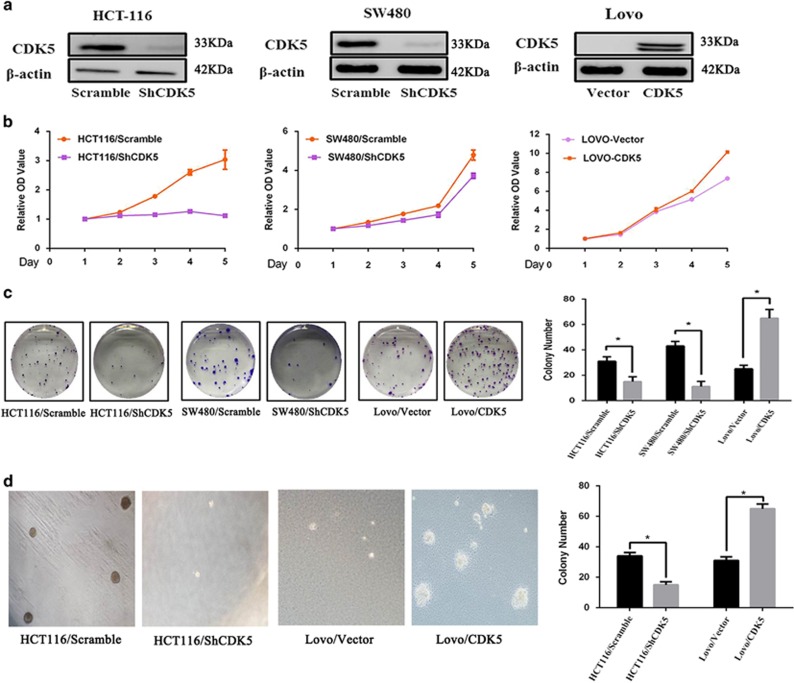
CDK5 promoted the aggressive behavior and proliferation ability of CRC cells *in vitro*. (**a**) Western blotting analyses of CDK5 in specific shRNA-transduced stable HCT116, SW480 cells and Lovo cell with ectopic expression of CDK5. (**b** and **c**) Proliferation assay of CCK8 experiments and colony-formation assays were used for detecting the proliferation ability in CDK5 knocked down or overexpression CRC cell lines. (**d**) Overexpression of CDK5 promoted cell growth of Lovo and silencing endogenous CDK5 inhibited the cell growth of HCT116 as determined by anchorage-independent growth-ability assays. Only cell colonies >0.1 mm in diameter were counted. *P<0.05

To investigate the biological function of CDK5 in CRC cells, first we established stable CDK5 silencing cell lines using a specific shRNA in SW480, HCT116 cell lines and CDK5 overexpressing CRC cell lines with Lovo cell line (Figure 2a). Strikingly, knockdown of CDK5 exhibited a remarkable inhibition of cell proliferation ability and cell colony formation (Figures 2b and c). Besides, knockdown of CDK5 in HCT116 caused obvious reduction in the colony number and colony size in soft agar (Figure 2d). Interestingly, we also preformed a pharmacological inhibition of CDK5 in HCT116 and SW480 cell lines with a specific CDK5 inhibitor roscovitine (ROS) and we found that treatment of ROS can dramatically decreased the ablitity of CRC cells in cell survival and migration (Supplementary Figure S1).

Conversely, overexpression of CDK5 promoted an aggressive phenotype of the Lovo cell line by enhancing its cell proliferation ability, colony-formation density and soft agar colonies. (Figures 2b–d).

We also investigate the effects of CDK5 deregulation on cell cyle progression using flow cytometric analysis. The results demonstrated that depletion of CDK5 in CRC cell lines arrested HCT116 and SW480 cells at the G1/S-phase transition, whereas overexpression of CDK5 in Lovo cells accelerated the G1- to S-phase transition (Supplementary Figure S2).

### CDK5 promoted metastasis of CRC *in vitro* and *in vivo*

**Figure 3:**
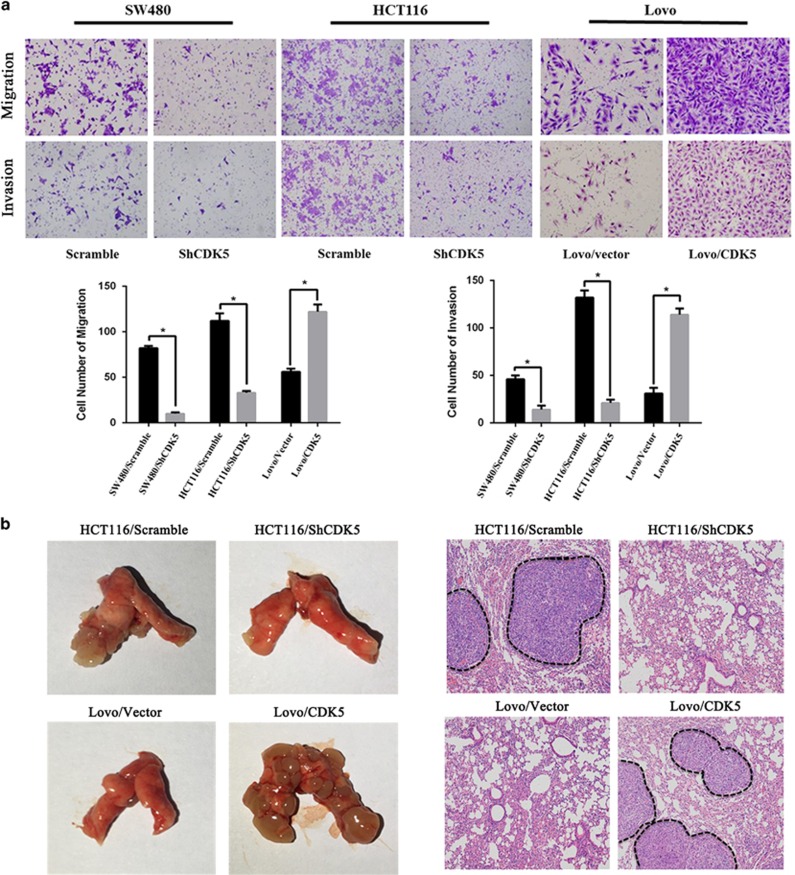
CDK5 promoted metastasis of CRC *in vitro* and *in vivo*. (**a**) Transwell assays were performed to detect the migration and invasion ablilty of CRC cell lines with CDK5 knocked down or overexpressed, respectively. Error bars represent mean±S.D. from three independent experiments. *P<0.05. (**b**) Representative images of gross specimens and hematoxylin and eosin staining of lung metastatic lesions formed in mice orthotopically implanted with HCT116/Scramble, HCT116/ShCDK5, Lovo/Vector and Lovo/CDK5 cell lines

We then evaluated the effect of CDK5 on the metastasis of CRC cell lines. Results from transwell assays revealed that knocking down of CDK5 decreased migration and invasion ability of CRC cells, while overexpression of CDK5 achieved the opposite effect (Figure 3a). Furthermore, we also achieved the same results in *in vivo* metastasis assays. As shown in Figure 3b, tumor cells formed by knocking down of CDK5 cells showed weaker metastatic ability and formed less tumors in the lungs, while tumors cells formed by CDK5-overexpressing cells were more invasive to form metastatic tumors in the lungs of nude mice. These data strongly suggested that CDK5 was involved in enhancing the metastatic capacity of CRC.

### Knocking down CDK5 induced differential expression of genes involved in cellular proliferation, survival and movement and downregulated a cluster of genes of ERK5–AP-1 axis

**Figure 4:**
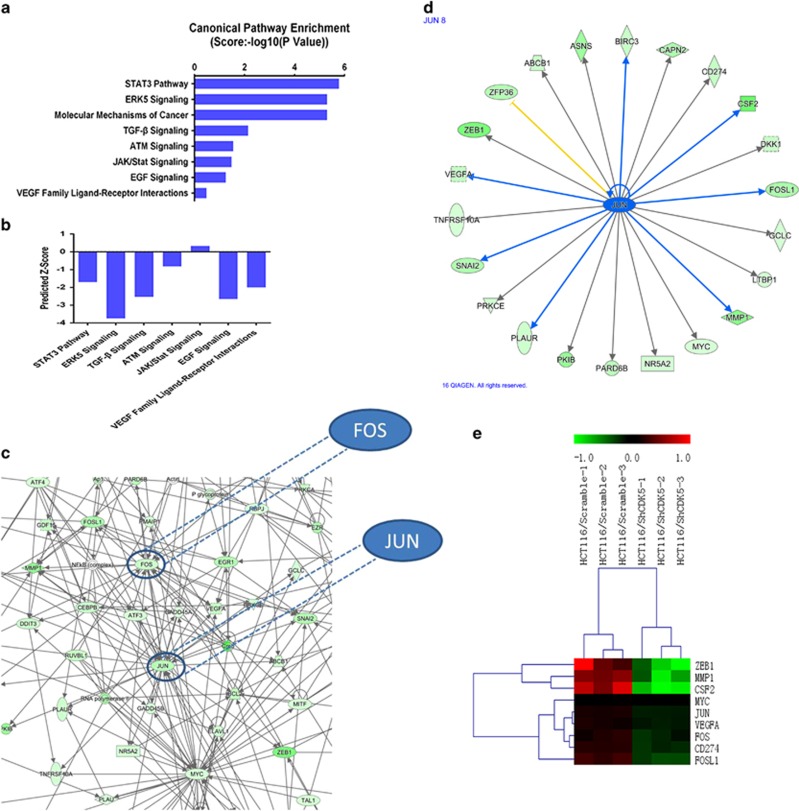
Knocking down CDK5 downregulated a cluster of genes of the ERK5–AP-1 axis. (**a** and **b**) Canonical pathway enrichment of whole-genome expression microarray in CDK5 knocked down HCT116 cells was analyzed by IPA. Predicted activation state of each pathway was based on the regulation *z*-score (Increased: The *z*-score is ⩾2. IPA predicts that the process or disease will increase. Decreased: The *z*-score ⩽−2. IPA predicts that the process or disease will decrease). (**c** and **d**) Gene interaction network suggested a cluster of genes regulated by transcription factors FOS and JUN (In IPA database, JUN was used to represent transcription factor AP-1). (**e**) Heat-map analysis showed a cluster of AP-1-dependent genes regulated by CDK5 as indetified by microarray profiling

To further understand the underlying molecular mechanisms of CDK5 mediating carcinogenesis in CRC, we performed whole-genome expression microarray in HCT116/Scramble and HCT116/ShCDK5 cells. According to the analysis of Ingenuity Pathway Analysis (IPA) database, knocking down CDK5 remarkably downregulated a series of genes that involved in celluar proliferation, survival and movement (Supplementary Figure S3). Besides, knocking down CDK5 significantly repressed several key carcinoma molecular pathways (Figure 4a), with the ERK5 signaling pathway mostly inhibited (*z*-score: −3.74, Figure 4b). Furthermore, the gene interaction network showed that the downsteram molecular factors of ERK5 signaling pathway, FOS and JUN, which had also been the key elements of the transcriptional factor AP-1, had a central role in the regulation of a cluster of genes related to cell proliferation and migration (Figures 4c–e, the fold change is shown in Supplementary Table S3).

### CDK5 activated the ERK5–AP-1 signaling axis via regulating the phosphorylation level of ERK5

**Figure 5:**
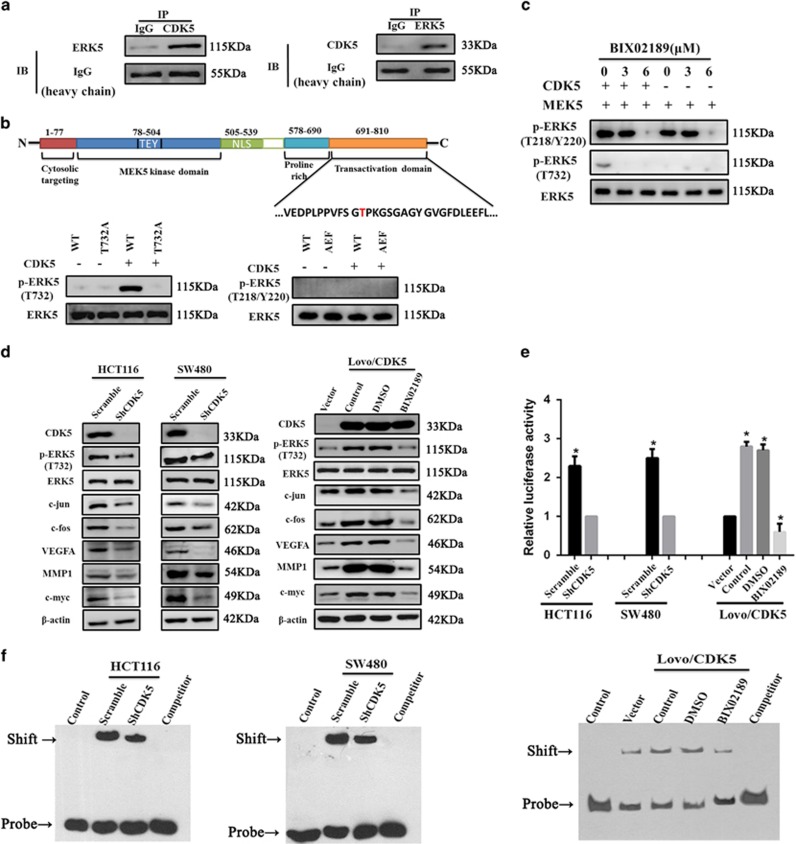
CDK5 directly phosphorylated ERK5 at Thr732 and modulated the ERK5–AP-1 signaling axis. (**a**) Co-immunoprecipitation revealed the relationship between CDK5 and ERK5 in non-transfected HCT116 cells. (**b**) *In vitro* kinase assays showed the specific site of ERK5 that CDK5 directly targeted. (**c**) *In vitro* kinase assays revealed the effect of phosphorylation inhibition about two ERK5's phosphorylation site (Thr732, Thr218/Tyr220) under various considerations of ERK5 inhibitor BIX02189 (0, 3, 6 *μ*M). (**d**) Whole proteins from CDK5 knocked down cell lines and Lovo/CDK5 cells, which were treated with the ERK5 inhibitor BIX02189 (3 *μ*M) or DMSO for 24 h, were used for detecting the indicated proteins of the ERK5–AP-1 axis by western blotting. (**e**) Activity of the AP-1 luciferase report gene in the indicated cells described above are shown. (**f**) EMSA assays about the AP-1 activity in the indicated cells described above are shown

Next we investigated whether CDK5 was involed in the regulation of ERK5–AP-1 signaling axis in CRC. Co-immunoprecipitation revealed that CDK5 could directly interact with ERK5 in non-transfected HCT116 cells (Figure 5a). Besides, point-mutation studies and *in vitro* kinase assay showed that CDK5 directly phosphorylate ERK5 at Thr732 but not the canonical site of ERK5 at TEY microdomain (Figure 5b). Furthermore, this phosphorylation phenomenon could be specifically inhibited when treated with ERK5-specific inhibitor BIX02189 at the concerntration of 3 *μ*M (Figure 5c).

We performed more experiments in stably transfected cell lines. Cell immunofluorescence assay showed that CDK5 enhanced the expression level of p-ERK5 with more nuclear accumulation (Supplementary Figure S4). Consistently, as shown in Figure 5d and Supplementary Figure S5, downregulation of CDK5 reduced the phosphorylation level of ERK5 at Thr732 and protein level of c-fos, c-jun and some of their important target genes, such as VEGFA, MMP1 and c-myc. On the contrary, overexpression of endogenous CDK5 significantly increased the phosphorylation level of ERK5 at Thr732 and the level of c-fos, c-jun, VEGFA, MMP1 and c-myc. Also, when treated with BIX02189 at the conceration of 3 *μ*M in CDK5-overexpressed Lovo cells, the phosphorylation level of ERK5 at Thr732 and proteins described above were significantly reduced compared with the control group treated with dimethyl sulfoxide (DMSO; Figure 5d). Also, electrophoretic mobility shift assay (EMSA) experiment and luciferase assay showed that knockdown of CDK5 reduced the AP-1's DNA-binding ability and the luciferase reporter gene activity of AP-1, while overexpression of CDK5 significantly increased the AP-1's DNA-binding ability and its luciferase reporter gene activity, except treated with BIX02189 in Lovo/CDK5 cells (Figures 5e and f).

### The CDK5–ERK5–AP-1 signaling axis could promote CRC carcinogenesis both *in vitro* and *in vivo*

**Figure 6:**
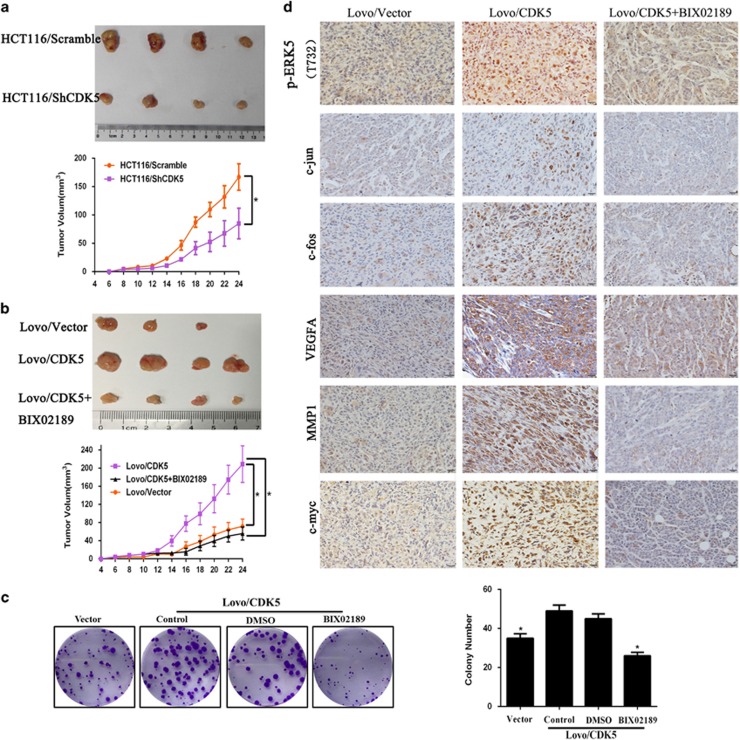
CDK5–ERK5–AP-1 signaling axis could promote CRC carcinogenesis both *in vitro* and *vivo*. (**a** and **b**) Representative examples of tumors in nude mice injected with the indicated cells. (**c**) Lovo/CDK5 cell proliferation was determined by colony-formation assay after treatment with BIX02189 or DMSO. Error bars represent mean±S.D. from three independent experiments. *P<0.05. (**d**) IHC staining for ERK5–AP-1 axis in CRC xenografts. Scale bars: 20 *μ*m

To confirmed the effect of CDK5–ERK5–AP-1 signaling axis on CRC carcinogenesis, we performed tumorigenesis assays in nude mice. Results from Figures 6a and b showed that tumors formed by CDK5-silenced cells were smaller and lighter in weight, while tumors formed by CDK5-transduced CRC cells grew more rapidly and were larger in size. All mice injected with Lovo/CDK5 cells generated tumors (4/4), whease only three mice in the control group formed tumors (3/4). Noteworthily, after treated with BIX20189, tumors grew slowly and size of them were smaller than that of the untreated groups (Figure 6b), which was consisted with the colony formation assay showing that the growth ability of LOVO/CDK5 cells treated with BIX02189 was inhibited significantly compared with the control cells (Figure 6c). Also, IHC staining also showed the similar results that CDK5 promoted the expression level of ERK5–AP-1 *in vivo* (Figure 6d).

### Clinical relevance of CDK5–ERK5–AP-1 axis in human CRC

**Figure 7:**
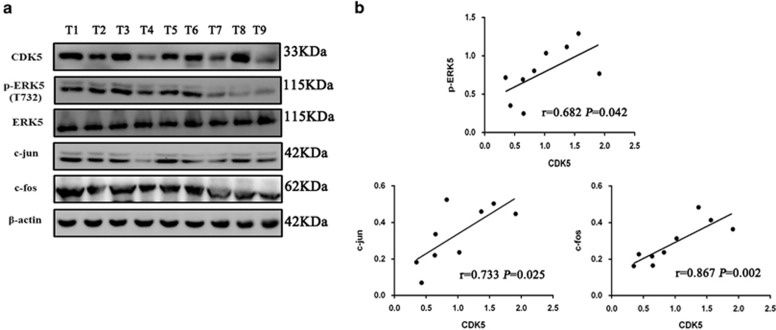
Clinical relevance of CDK5–ERK5–AP-1 axis in human CRC. (**a**) Western blotting analysis of CDK5, p-ERK5 (Thr732), c-jun and c-fos in nine fresh human CRC samples(T1–T9); *β*-actin and total ERK5 were used as loading controls. (**b**) Correlation analysis were shown by Spearman's correlation analyses between CDK5 expression and p-ERK5, c-jun and c-fos

We further investigated whether CDK5 expression and ERK5–AP-1 activation are clinically relevant in human CRC. As shown in Figure 7, the level of CDK5 expression in 9 freshly collected clinical CRC samples significantly correlated with the phosphorylation level of ERK5 at Thr732 (*r*=0.683 *P*=0.042), c-jun (*r*=0.732 *P*=0.025) and c-fos (*r*=0.867 *P*=0.002). These data further supported the notion that CDK5 could activate the ERK5–AP-1 signaling axis and lead to a poor clinical outcome in CRC.

## Disscussion

Although CDK5 protein level was previously observed in CRC,^15^ this is the first study to investigate the clinicopathological significance of CDK5 in CRC. Our research has showed that high expression of CDK5 protein is significantly correlated with the aggressive characteristics (AJCC stage, tumor size, tumor differentiation and nodal metastasis) as well as poor survival of patients. Besides, a CRC microarray cohort from TCGA also verified our results. Similar to our results, in cancer such as lung cancer and pancreatic cancer, CDK5 and p35 overexpression were frequently but not solely associated with gene mutation.^10, 16^ These researches indicated that CDK5 may serve as a universal tumor biomarker to identify patients with poor clinical outcomes.

CDK5 was discovered in the early 1990 as proline-directed serine/threonine kinase in the CNS and emerged as a crucial regulator of neuronal development, function and disease.^5^ Recently, CDK5 has been proposed to possess other functions than that in nervous system, especially in cancer progression. Interestingly, CDK5 may have different roles during malignant development. Majority of studies supported that CDK5 might have a role in promotion of multistep process of tumor formation and progression, including transformation, proliferation, angiogenesis, invasion and metastasis.^17, 18^ On the contrary, Cao *et al.*^19^ found that CDK5 was decreased in gastric cancer and overexpression of CDK5 could inhibit gastric tumorigenesis. Our research suggested that different levels of CDK5 and its kinase activity were detected in CRC cells and knocking down CDK5 would be associated with lower proliferation, metastasis ablility and arrest G1–S transition of CRC cells, while overexpression of CDK5 performed the opposite effect. Thus these observations suggested that CDK5 might function as a tumor promotor in human CRC.

To further determine the mechanism as to how CDK5 promoted CRC cell proliferation and metastasis, we performed whole-genome expression microarray in CDK5 knocked down CRC cells. As expected, CDK5 controlled a cluster of genes and signaling pathways related to cancer proliferation and metastasis. Based on the analysis of IPA database, ERK5 signaling pathway ranked the top as the most highly inhibited and two of its downstream effectors, FOS and JUN, which comprised a key transcription factor activator protein 1 (AP-1), controlled a cluster of key oncogenic gene's expression. These data suggested a connective link between CDK5 and ERK5–AP-1 signaling axis. It is worth mentioning that data from genomic microarray showed that downregulation of CDK5 caused a series of gene alteration that belong to the downstream of ERK5 but not ERK5 itself, which mean that CDK5 might modulate ERK5 in a structurally modified manner at the protein level but not solely at the genetic level.

ERK5 is an effector kinase of the canonical three-tiered MAPK signaling cascade comprising MEKK2/3, MEK5 and ERK5 itself.^20^ During the past decade, the function of ERK5 signaling in cancer progression has been a hot research area. For example, inactivation of MEK5/ERK5 signaling pathway may reduce the expression of c-myc in CRC cells and finally supress cell proliferation, colony formation and cell invasion of CRC.^21^ A recent research also suggests that ERK5 signaling rescues intestinal epithelial turnover and tumor cell proliferation upon ERK1/2 abrogation, while a combination of MEK1/2 and ERK5 inhibitors show a more remarkable effect in inhibiting CRC cell growth.^22^

Interestingly, among the downstream molecular effectors of ERK5 signaling, phosphorylation of ERK5 in its TEY microdomain area or c-terminal region with multiple sites, such as Ser706 and Thr732, is able to activate transcription not only by direct phosphorylation of transcription factors but also by acting itself as a transcriptional coactivator for one of its key downstream effector, the AP-1 transcription factor.^23, 24^ AP-1 possesses a specific phenotype that consists of either a Jun–Fos heterodimer or a Jun–Jun homodimer.^25^ AP-1 has been demonstrated to have a critical role in tumor promotion via modulating the genetic transcription of some important oncogenes, such as VEGFA, MMP1, c-myc and so on.^26, 27, 28^ Previous studies revealed that AP-1 could promote the proliferation ability and enhance tumorigenic properties of CRC cells.^29, 30^ These researches suggest that the ERK5-AP-1 axis is a key signaling pathway in CRC progression, which is still needed more attention.

As expected, our data from both the *in vitro* and *in vivo* levels showed that CDK5 could modulate the ERK5–AP-1 axis. Although CDK5 has been found to closely link to some other MAPK signaling cascades in neurons,^31, 32^ it is the first time to illustrate the relationship between CDK5 and ERK5 pathway. We found that CDK5 could directly phosphorylate ERK5 at Thr732, which suited the conservative sequence of CDK5(X-S/T-P-X).^33^ Further studies showed that knockdown of CDK5 decreased the phosphorylating level of ERK5 at Thr732, AP-1 and some of its target genes' expression, which was reversed when CDK5 was overexpressed without a ERK5 inhibitor in CRC cells. As a result, when CDK5 activated the ERK5–AP-1 axis, some important oncogenic genes, such as VEGFA, MMP1 and c-myc, were deregulating, which finally resulted in the malignant development of human CRC both in *in vitro* and *in vivo* experiments. On the other hand, the CDK5–ERK5–AP-1 axis showed a significant clinical relevance in human CRC. Thus it is clear that CDK5 may promote cell proliferation and metastasis partly by modulating the ERK5–AP-1 axis.

In conclusion, our findings help provide evidence that elevated CDK5 in human CRC has an important role in the acquisition of the progression as well as poor prognosis of the tumor. Furthermore, we also identified the CDK5–ERK5–AP-1 axis as a potential oncogenic pathway in CRC. This study not only further elucidates the precise role of CDK5 in the pathogenesis of CRC but also provides new insight into the biological basis of cancer progression and may help to contribute to more effective therapeutic strategies for CRC.

## Materials and Methods

### CRC cell line and cell cluture

Human CRC cell lines (Caco-2, HCT116, SW480, SW620, Ls174t, Lovo and HT-29) were originally purchased from American Type Culture Collection (ATCC, Manassas, VA, USA). The cells were cultured in Dulbecco's modified Eagle's medium (Invitrogen, Carlsbad, CA, USA) supplemented with 10% FBS (HyClone, Logan, UT, USA) and 1% penicillin/streptomycin (Invitrogen).

### Human CRC tissue samples

Human CRC tissues and corresponding normal colon tissues for immunoblotting (freshly frozen) were provided by patients with CRC who had undergone surgical rsection in the Department of General Surgery, Nanfang Hospital, First Affiliated Hospital of Southern Medical University, Guangzhou, China. All patients provided written informed consent.

### Establishment of stable cell lines

The CDK5 shRNA sequence was sense 5′-TATAAGCCCTATCCGATGT-3′ and the control shRNA sequence was sense 5′-TTCTCCGAACGTGTCACGT-3′. The human CDK5 construct was generated by cloning PCR-amplified full-length human CDK5 cDNA with the transcript NM_004935. Both the shRNA sequence and the CDK5 construct were subcloned into lentiviral expression vector by GeneChem (Shanghai, China). The shRNA lentivirus and the control lentrvirus were transfected into the relative high expressing CDK5 cell lines HCT116 and SW480. The CDK5 low expressing cell lines Lovo was transfected with lentiviruses expressing CDK5 cDNA.

### TMA and IHC

The TMA containing a total of 89 colon cancer patients, together with the data of pathological staging in accordance with TNM classification of the AJCC 2010 and overal survival time for all cases (Supplementary Table S1), was obtained from the National Engineering Center For Biochip at Shanghai, China. The IHC staining of CDK5 with scoring were carried out as previously described.^34^ The section of TMA was baked at 65 °C for 30 min. Then the section were deparaffinized with xylenes and rehydrated. After treatment with 3% hydrogen peroxide in methanol to quench the endogenous peroxidase activity, the section were submerged into citrate buffer and high-pressure boiled for antigenic retrieval, followed by incubation with 1% bovine serum albumin to block the nonspecific binding. Rabbit anti-CDK5 (1 : 100; Abcam, Cambridge, MA, USA) or rabbit anti-p35 (1 : 100; Cell Signaling Technology, MA, USA) was incubated with the section overnight at 4 °C. For negative controls, the rabbit anti-CDK5 or anti-p35 antibody was replaced with normal goat serum. After washing, the TMA section were treated with anti-rabbit secondary antibody (Zhongshan Biotech, Beijing, China). The tissue section were incubated with 3,3-diaminobenzidin and counterstained with hematoxylin, dehydrated and mounted. The section were reviewed and scored independently by two observers, based on both the proportion of positively stained tumor cells and the intensity of staining. The proportion of positive tumor cells was scored as follows: 0 (no positive tumor cells), 1 (<10% positive tumor cells), 2 (10–50% positive tumor cells), and 3 (>50% positive tumor cells). The intensity of staining was graded according to the following criteria: 0 (no staining); 1 (weak staining=light yellow), 2 (moderate staining=yellow brown), and 3 (strong staining=brown). The staining index was calculated as staining intensity score × proportion of positive tumor cells. Using this method of assessment, the expression of CDK5 was scored as 0, 1, 2, 3, 4, 6 and 9. Cutoff values for CDK5 were chosen on the basis of a measure of heterogeneity by log-rank test statistical analysis with regard to overall survival. An optimal cutoff value was identified: the score of ⩾4 was used to define tumors as high CDK5 expression and ⩽3 as low expression of CDK5.

### Western blotting analysis

Western blotting was performed as previously described,^35^ using anti-CDK5, anti-FAK, anti-p-FAK (Ser732), anti-PAK1, anti-p-PAK1 (Thr212), anti-ERK5, anti-p-ERK5 (Thr218/Tyr220) (Abcam), anti-p-ERK5 (Thr732), anti-p35 (Cell Signaling Technology, Danvers, MA, USA; anti-p-ERK5 Thr732 was custom-made from CST), anti-c-fos, anti-c-jun (Bioworld Technology, Louis Park, MN, USA), anti-c-myc, anti-VEGFA and anti-MMP1 antibodies (Proteintech, Chicago, IL, USA). Loading control was used with a mouse anti-*β*-Actin monoclonal antibody (Proteintech).

### Cell proliferation assay

The stable cell lines were seeded on 96-well plates at initial density of (1–2 × 10^3^/well). At each time point, cells were detected by the Cell Counting Kit-8 Kit following the kit assay protocol. Besides, the sensitivity of HCT116 and SW480 to CDK5-specific inhibitor ROS was also determined by CCK8 assay at various concentrations of ROS. The same concentrations of DMSO were used as control.

### Colony-formation assays

Cells were seeded on a six-well plate (200 cells/well) and incubated at 37 °C in a humidified 5% CO_2_ atmosphere incubator. After 2 weeks, cells were fixed and stained with hematoxylin. Colonies containing >50 cells were counted. Three independent experiments were performed for each cell line.

### Soft agar assay

Four hundred cells were suspended in 2 ml complete medium containing 0.35% agar (Sigma, CA, USA). Then the agar–cell mixture was plated on top of a bottom layer with 0.7% complete medium agar mixture. Twelve days later, the colonies were measured with an ocular micrometer (Olympus, Tokyo, Japan); colonies > 0.1 mm in diameter were counted. The experiment was repeated independently three times for each cell line.

### Cell cycle analysis

The effect of CDK5 on cell cycle progression was determined by flow cytometry using a Cell Cycle Detection Kit (KeyGEN BioTECH, Nanjing, China). The transfected cells were seeded in six-well plates at 2 × 10^5^ cells/well and cultured for 2 days. Cells were then collected and fixed with cold 70% ethanol for 24 h at 4 °C. Next, cells were incubated with 0.5 mg/ml of propidium iodide along with 0.1 mg/ml of RNase A. Cell cycle analysis was performed using flow cytometry (BD, San Diego, CA, USA).

### Cell migration and invasion assays

For migration assays, the transwell chambers equipped with 8-*μ*m membranes were used (Corning Incorporated, Corning, NY, USA). For invasion assays, The membrane was covered with 40 *μ*l of the BD Matrigel (diluted 1 : 8 with serum-free medium) in advance. The stable cells (1–1.5 × 10^5^) were seeded into the transwell chambers with serum-free medium while the lower chamber was covered with 500 *μ*l medium supplemented with 20% fetal calf serum. After 48 h incubation at 37 °C, cells were harvested and fixed in 4% paraformaldehyde for 20 min and then stained with 0.5% crystal violet diluted in methanol for 25 min. The membrane was removed and mounted onto glass slides and counted (five × 200 random fields per membrane). Three independent experiments were performed and the data are presented as mean±S.D.

### Co-immunoprecipitations

Co-immunoprecipitations of endogenously expressed proteins were performed in HCT116 cells. In all, 5 × 10^6^ cells were lysed using ice-cold RIPA buffer containing protease and phosphatase inhibitors. After a 30-min incubation at 4 °C, total extracts were clarified by centrifugation at 12 000 r.p.m. for 30 min. Then the cell extracts were precleared with 50 *μ*l protein A+G agarose (7seaBiotech, Shanghai, China) for an hour at 4 °C, and the cleared extracts were immunoprecipitated with 2 *μ*g of the indicated antibodies (anti-IgG, anti-CDK5 and anti-ERK5) and 50 *μ*l protein G-agarose overnight at 4 °C. The immune complexes were washed three times with PBS buffer for 3 min each time. After the third wash, immunoprecipitates were re-suspended in SDS-PAGE sample buffer containing loading dye and used for western blotting as previously described.^35^

### Mutant generation of ERK5 proteins and *in vitro* kinase assay

Mutatgenesis of ERK5 was generated by Genesent (Wuhan, China). Briefly, the indicated amino acids were changed as follows: ERK5^T732A^ (Thr732 to Ala), ERK5^AEF^ (Thr218 to Ala, Tyr220 to Phe). In addition, a construct containing wild-type ERK5 sequence was generated. All the versions were tagged with an HA epitope into a pBOBI lentivirus vector.

For the *in vitro* kinase assay, human 293 cell lines transfected with above lentivirus vectors were treated with BIX02189 under the indicated conditions, and then the cells were lysed using ice-cold RIPA buffer containing protease and phosphatase inhibitors and immunoprecipitated with anti-HA Mab-sepharose (7seaBiotech) and anti-HA antibody (Abcam) overnight at 4 °C. After washing three times with PBS, the eluted immunoprecipitates were incubated with 10 ng recombinant active CDK5/P35 protein or MEK5 protein (Millipore, Billerica, MA, USA) in kinase buffer (25 mMTris (pH 7.5), 2 mM DTT, 5 mM *β*-glycerolphosphate, 1 mM Na_3_VO_4_ and 10 mM MgCl_2_) along with 5 *μ*M ATP for 30 min at 37 °C. Reactions were terminated by the addition of 20 *μ*l 5 × loading buffer, and the samples were then resolved by immunoblotting. Phosphorylation of Thr732 and Thr218/Tyr220 of ERK5 was detected by corresponding specific phospho-antibodies.

### Cell immunofluorescence assay

CRC cells were seeded in 12-well plates for 48 h. Next the cells were fixed in 4% paraformaldehyde and permeabilized in 0.5% Triton at 37 °C for 30 min. After blocking for 1 h with 5% BSA, the cells were incubated overnight with anti-p-ERK5 antibody (Bioss, Beijing, China). Cell incubated with anti-p-ERK5 antibody were then incubated at 37 °C with FITC-conjugated anti-rabbit IgG (Zhongshan Biotech). Then the cells were stained with DAPI. Images were captured under a fluorescence microscope. The experiments were performed at least three times.

### Electrophoretic mobility shift assay

EMSA was performed as previous study using the LightShift Chemiluminescent EMSA Kit (Beyotime, Shanghai, China).^36^ Biotin-labeled and unlabeled probes as competitor containing AP-1-specific binding were designed as the following sequence :5′-CGCTTGATGACTCAGCCGATCTGACTCA-3′ (forward); and 5′-TGAGTCAGATCGGCTGAGTCATCAAGCG-3′ (reverse).

### Dual-luciferase reporter assay

Cells (3 × 10^4^) were seeded in triplicates in 48-well plates and allowed to settle for 24 h. In all, 0.3 *μ*g of REPOTM-AP-1-luc plasmid, or the control-luciferase plasmid, plus 30 ng of pGMR TK renilla plasmid (GenomeDitech, Shanghai, China) were transfected into CRC cells using the Lipofectamine 3000 reagent (Invitrogen). Forty-eight hours after transfection, luciferase and renilla activities were measured using the Dual Luciferase Reporter Assay Kit (Promega, Madison, WI, USA).

### Microarray analysis

The microarray experiments were performed at Shanghai Biotechnology Corporation, Shanghai, China. Total RNA was isolated using the RNase Kit (Takara Bio, Dalian, China) from three replicate samples of the stable cell lines HCT116/Scramble and HCT116/ShCDK5. Agilent Whole Human Genome Oligo Microarray(4 × 44K) (Agilent Technologies, Santa Clara, CA, USA) was used for transcriptome analysis as previously described.^37^ Processed signals were normalized by the GeneSpring Software GX 12.6.1(Agilent Technologies) and more details were shown in GSE81085. To assess the molecular signaling pathway change after knockdown of CDK5, data analysis, including canonical pathway and gene network interaction, was performed by the IPA. The *P*-value was calculated via the Ingenuity Pathways Knowledge Base-depedent Fisher's exact test.

### *In vivo* metastasis assay

To evaluate the metastatic potential to lung of cancer cells *in vivo*, 2 × 10^6^ HCT116/Sramble, HCT116/ShCDK5, Lovo/Vector and Lovo /CDK5 cells were injected into the tail vein of 4–6-week-old Balb/C athymic nude mice (Animal Center of Southern Medical University, Guangzhou, China; nu/nu; *n*=3 for each group). All mice were housed and maintained under specific pathogen-free conditions. Two months later, the mice were killed and the lungs of them were dissected for futher analysis.

### Tumorigenesis in nude mice

Xenograft tumors were generated by subcutaneous injection of CRC cells (5 × 10^6^), including Lovo/Vector and Lovo /CDK5, HCT116/Sramble and HCT116/ShCDK5, on the right hind limbs of 4–6-week-old Balb/C athymic nude mice (nu/nu; *n*=4 for each group). All mice were housed and maintained under specific pathogen-free conditions. When tumors grew in one group injected with Lovo/CDK5 cells, BIXO2189 were used to inject at the positions of tumors (100 *μ*l; solvent: DMSO/PEG/Tween80/ddH_2_O: 1 : 15 : 2.5 : 31.5; 20 mg/kg/3days (translating from the cellular level)). The tumor volume was calculated using the following formula: tumor volume=4π/3 × (width/2)^2^ × (length/2), where the width and length were the shortest and longest diameters, respectively. After 3–4 weeks, the mice were killed during 21–30 days and tumors were then dissected and used for IHC staining as mentioned above (concentrations of different antibodies used for IHC staining are: anti-pERK5(Thr732):1 : 200; anti-c-fos:1 : 200; anti-c-jun:1 : 100; anti-VEGFA:1 : 150; anti-MMP1:1 : 250; and anti-c-myc:1 : 100). All animal experiments were approved by the Use Committee for Animal Care and preformed in accordance with the institutional guidelines.

### Statistical analysis

All statistical analyses were carried out using SPSS version 16.0 (SPSS Inc., Chicago, IL, USA). Pearson's chi-square was used to analyze the relationship between CDK5 expression and the clinicopathological features of CRC. Survival curves were plotted by the Kaplan–Meier method and compared using log-rank test. The Cox proportional hazard model was used to calculate relative risk ratios. Univariate and multivariate survival distributions were compared using log-rank test. Two-tailed independent Student's *t*-test was used for analyzing two groups. *P*<0.05 was considered significant.

## Figures and Tables

**Table 1:** Correlation between clinicopathological features and CDK5 expression levels

**Variables**	***n***	**CDK5 expression**		***χ***^**2**^ a	***P***
		**Low**	**High**		
*Gender*				0.32	0.572
Male	46	22	24		
Female	43	18	25		
					
*Age (years)*				0.103	0.748
<59	26	11	15		
⩾59	63	29	34		
					
*Tumor size (cm in diameter)*				10.156	**0.001**
<5	37	24	13		
⩾5	52	16	36		
					
*Nodal metastasis*				4.867	**0.027**
N0	58	31	27		
N1/2	31	9	22		
					
*Differentiation*				6.619	**0.034**
Well	21	14	7		
Moderate	55	23	32		
Poor	13	3	10		
					
*AJCC stage*				6.619	**0.01**
I/II	56	31	25		
III/IV	33	9	24		

a Pearson's chi-square was used for the assay. Significant at *P*<0.05

## Abbreviations

CDK: cyclin-dependent kinase
CRC: colorectal cancer
AJCC: American Joint Committee on Cancer
EMSA: electrophoretic mobility shift assay
IHC: immunohistochemistry
TMA: tissue microarray
HR: hazard ratio
