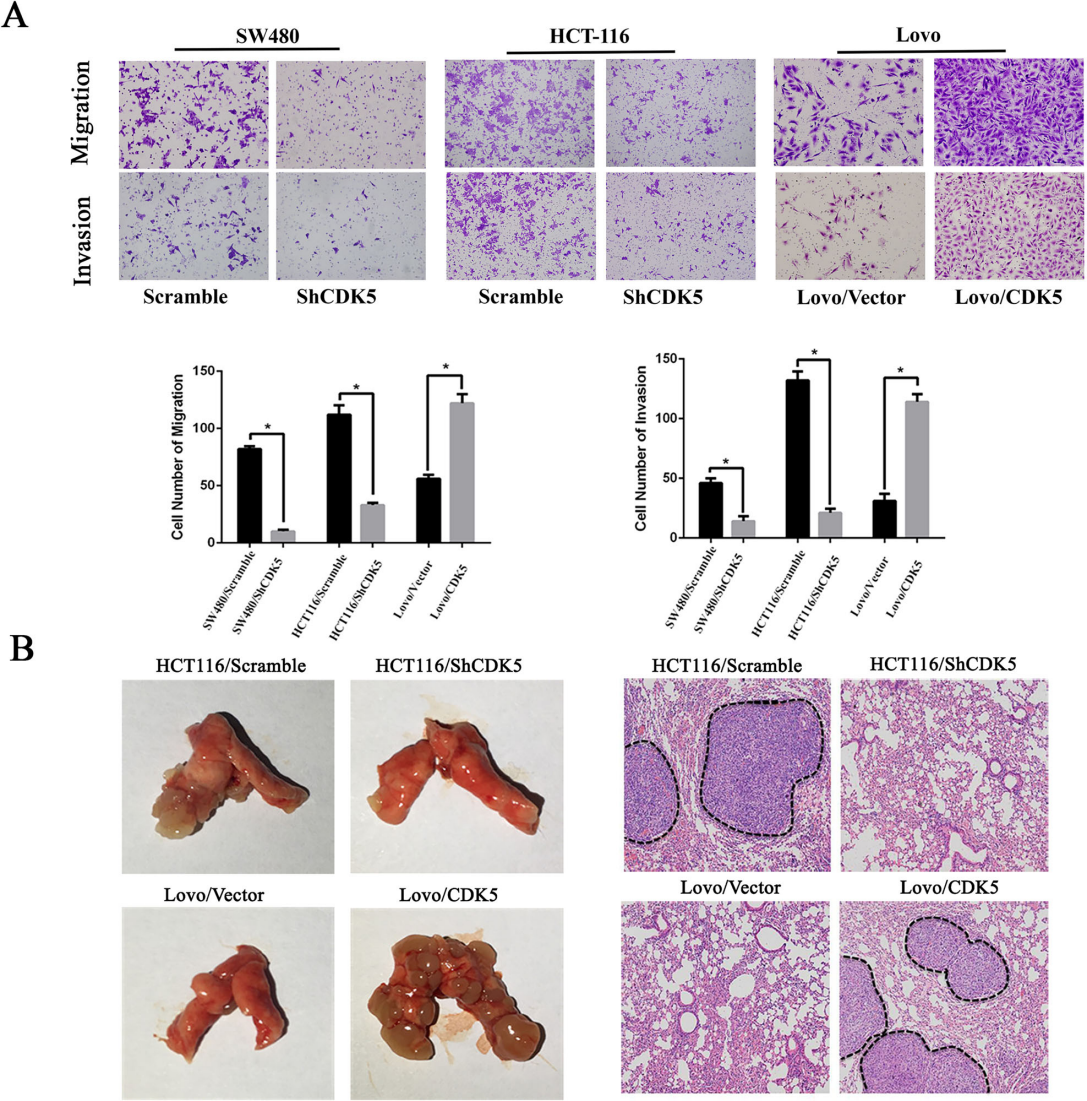# Correction: CDK5 functions as a tumor promoter in human colorectal cancer via modulating the ERK5–AP-1 axis

**DOI:** 10.1038/s41419-026-08780-4

**Published:** 2026-04-24

**Authors:** Kangmin Zhuang, Juchang Zhang, Man Xiong, Xianfei Wang, Xiaobei Luo, Lu Han, Yan Meng, Yali Zhang, Wenting Liao, Side Liu

**Affiliations:** 1 Guangdong Provincial Key Laboratory of Gastroenterology, Department of Gastroenterology, Nanfang Hospital, Southern Medical University, Guangzhou, Guangdong China; 2 Department of Gastroenterology, Affiliated Hospital of North Sichuan Medical College, Nanchong, Sichuan China; 3 Department of Pathology, Nanfang Hospital, Southern Medical University, Guangzhou, Guangdong China; 4 Department of Pathology, School of Basic Medical Sciences, Southern Medical University, Guangzhou, Guangdong China

Correction to: *Cell Death & Disease* 10.1038/cddis.2016.333, published online 13 October 2016

Careless use of images including loading controls of β-actin for SW480 and Lovo of Figure 2a and between invasion and migration images of Figure 3a.

**Original Figure 2** 
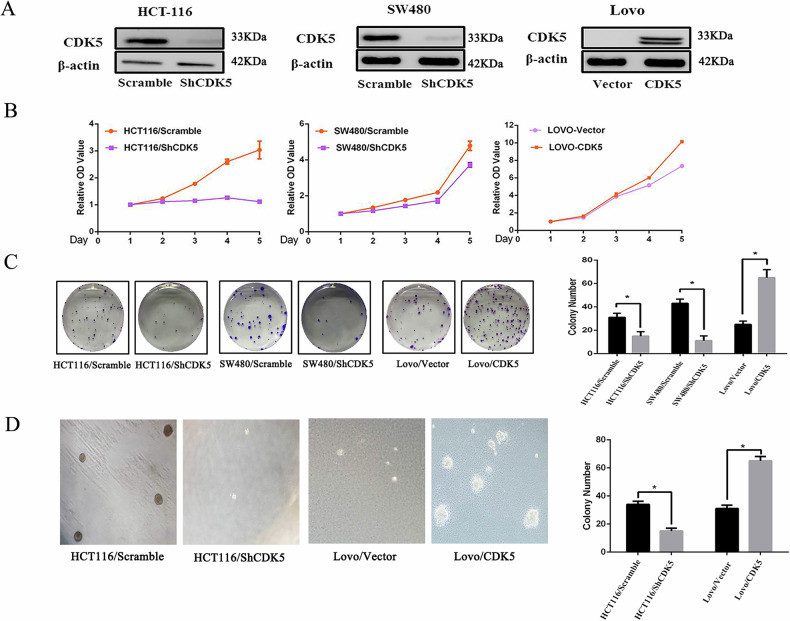


**Correction Figure 2** 
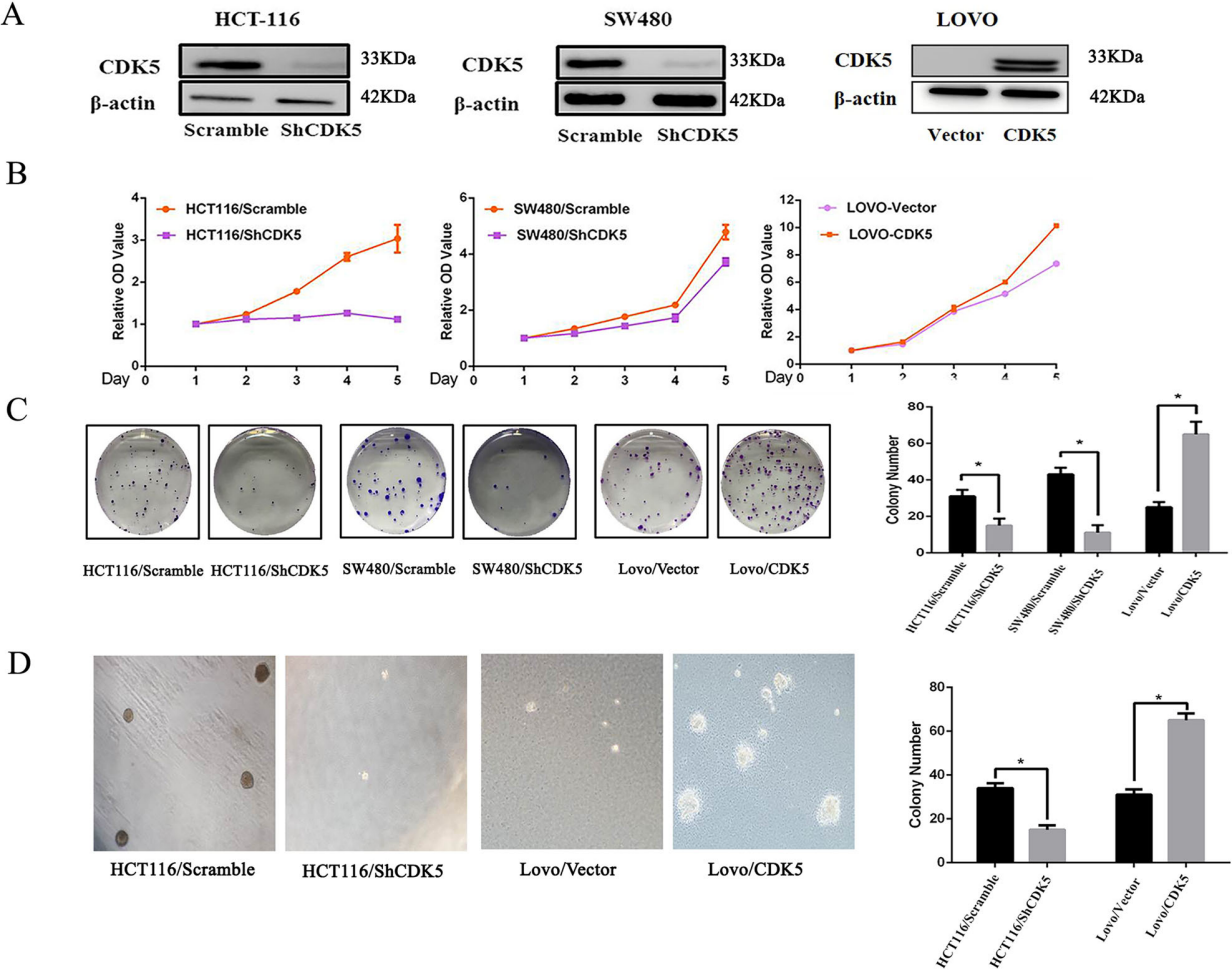


**Original Figure 3** 
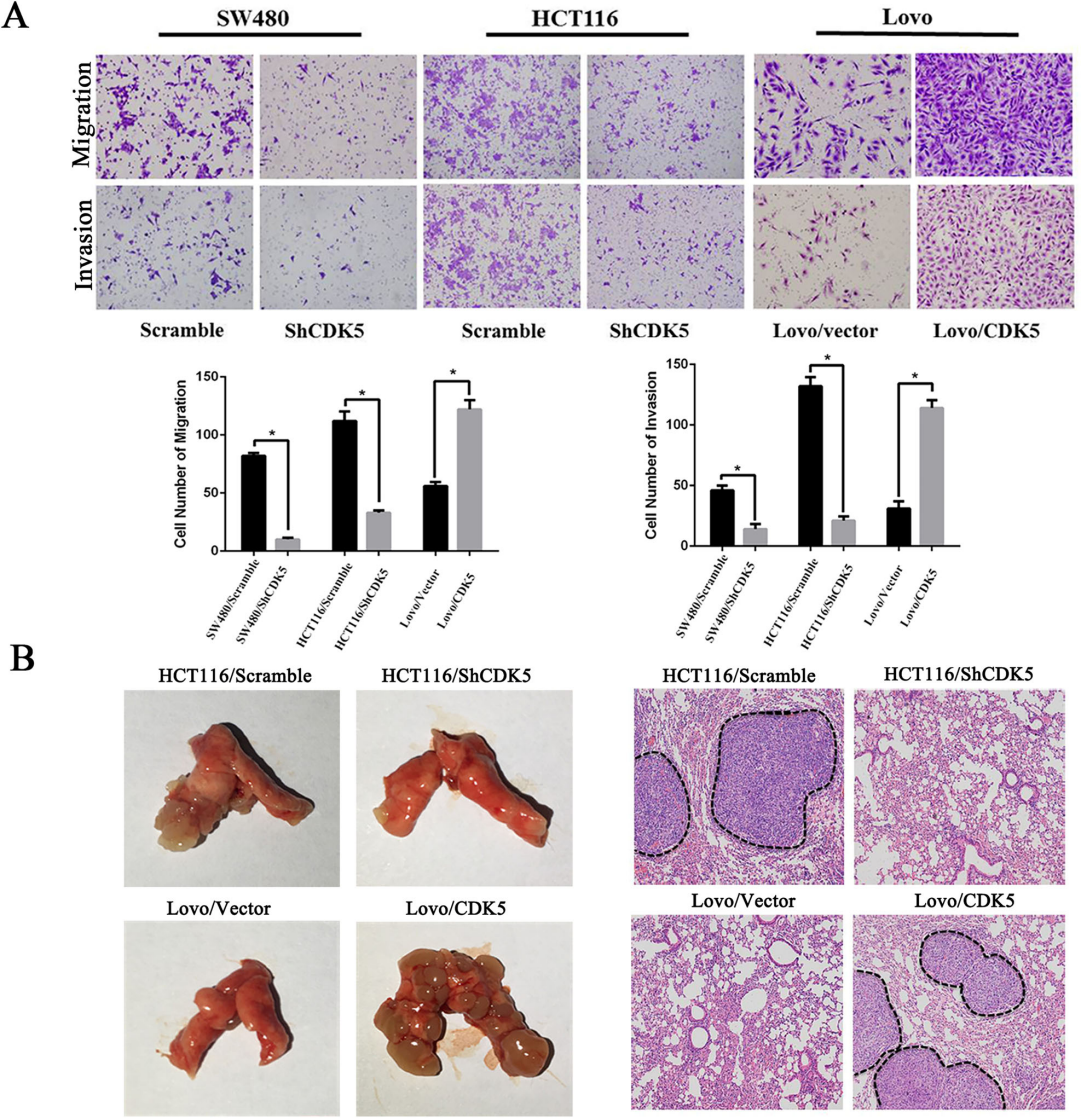


**Correction Figure 3**