# Supplementary material for: CDK5 functions as a tumor promoter in human colorectal cancer via modulating the ERK5–AP-1 axis
Source: Cell Death Dis. 2016 Oct 13;7(10):e2415–. doi: 10.1038/cddis.2016.333 (PMC5133995; doi:10.1038/cddis.2016.333)
Supplement: Supplementary Tables [file cddis2016333x1.pdf]

Table S1

The clinicopathological characteristics of 89 clinical samples in TMA

Sample ID	Status	Survival time (months)	Gender	Age (years)	Differentiation	Tumor size (cm)	AJCC stage	CDK5 score
1454	Alive	64	Male	72	Moderate	6.5	IIA	4
1455	Dead	1	Female	57	Moderate	6.5	IIIA	4
1456	Dead	13	Male	76	Moderate	5.5	IIA	4
1458	Alive	64	Female	63	Moderate	4.5	IIA	3
1461	Alive	63	Female	78	Moderate	7	IIIC	4
1462	Dead	22	Male	78	Moderate	8	IV	6
1464	Dead	7	Male	63	Poor	5.5	IIIC	4
1502	Alive	63	Female	68	Moderate	6	IIA	4
1503	Alive	63	Male	39	Moderate	6	IIB	2
1504	Dead	23	Male	68	Moderate	5.5	I	1
1505	Alive	63	Male	62	Moderate	2.5	IIA	3
1508	Alive	63	Male	78	Moderate	5	IIA	4
1510	Dead	44	Female	50	Moderate	4	IIB	6
1556	Alive	62	Male	73	Moderate	11	IIA	3
1557	Dead	38	Male	68	Moderate	6	IIB	6
1558	Dead	13	Female	87	Moderate	6	IIIC	9
1559	Dead	8	Female	52	Poor	7	IIB	6
1560	Alive	62	Female	51	Moderate	2.7	I	2
1561	Dead	56	Male	55	Moderate	3.5	IIIC	6
1562	Dead	17	Male	73	Poor	6.5	IV	9
1563	Alive	62	Female	61	Moderate	3	IIA	2
1564	Dead	12	Female	48	Poor	1.5	IIA	3
1565	Alive	62	Female	59	Moderate	3	I	2
1566	Dead	40	Female	77	Moderate	4	I	2

1567	Dead	42	Male	78	Moderate	5	IIIB	4
1570	Alive	62	Male	31	Poor	4	IIIB	3
1571	Dead	33	Female	79	Moderate	7	IIA	4
1572	Alive	61	Male	81	Moderate	4	IIIB	4
1573	Alive	61	Male	85	Moderate	4.3	IIA	2
1574	Dead	40	Male	90	Moderate	7	IIB	4
1576	Alive	61	Female	70	Moderate	4.5	I	2
1577	Dead	23	Female	66	Poor	5	IIIC	3
1579	Alive	61	Male	73	Moderate	3.5	IIA	3
1614	Alive	61	Female	54	Moderate	3.5	IIA	2
1615	Alive	61	Male	50	Well	4	I	4
1616	Alive	61	Female	74	Well	5	IIA	2
1617	Alive	61	Male	80	Moderate	8	IIA	4
1619	Alive	61	Female	65	Moderate	4	IIA	4
1620	Alive	61	Female	59	Moderate	4.5	IIA	1
1622	Alive	61	Male	79	Moderate	4	IIA	4
1624	Dead	13	Female	76	Moderate	3.5	IIIB	1
1625	Alive	60	Female	76	Moderate	8	IIA	2
1626	Dead	39	Male	63	Moderate	5	IIIB	6
1628	Dead	25	Female	76	Moderate	8	IIIB	9
1629	Alive	61	Male	56	Moderate	4	IIA	3
1630	Alive	60	Female	44	Moderate	8	I	6
1663	Dead	13	Male	73	Moderate	5.5	IIA	3
1668	Alive	60	Female	66	Moderate	7.5	IIIA	6
1669	Dead	1	Female	48	Moderate	8	IIA	6
1732	Alive	59	Male	79	Moderate	6	IIA	2
1733	Alive	59	Male	55	Poor	5	IIIB	1
1735	Alive	59	Female	65	Moderate	4	IIA	1

1740	Dead	7	Female	73	Moderate	8	IIIB	4
1741	Alive	59	Male	81	Moderate	8	IIIB	2
1742	Alive	59	Male	61	Moderate	4.5	I	6
1743	Dead	16	Male	65	Poor	6	IIIC	6
1744	Dead	16	Male	61	Poor	4	IV	4
1745	Alive	59	Male	80	Moderate	4	IIA	9
1756	Alive	58	Female	71	Moderate	3	IIIB	3
1758	Alive	58	Female	55	Poor	11	IIA	6
1762	Alive	58	Female	69	Moderate	8	IIB	3
1764	Alive	58	Female	80	Poor	6	IIIB	4
1765	Dead	21	Male	55	Moderate	4	IIB	3
1767	Alive	58	Male	83	Well	5	IIA	2
1811	Alive	57	Male	73	Well	7	IIB	2
1813	Alive	57	Female	82	Poor	4	IIIB	1
1814	Alive	57	Female	69	Moderate	2	I	6
1815	Alive	57	Female	46	Moderate	6	IIA	3
1819	Alive	57	Female	56	Moderate	4.5	IIA	4
1820	Alive	56	Female	78	Moderate	4.5	IIA	2
1836	Alive	56	Female	81	Moderate	6	IIB	1
1839	Alive	56	Male	73	Moderate	5	IIB	3
1841	Alive	56	Female	50	Moderate	6	IIA	6
1904	Dead	19	Female	27	Poor	4	IIIC	2
1907	Dead	35	Male	54	Moderate	5	IIA	6
1914	Alive	55	Female	77	Moderate	5	IIIB	3
1915	Alive	55	Female	55	Moderate	9	IIIB	4
1917	Alive	55	Male	66	Moderate	2.7	I	6
1918	Dead	42	Male	60	Moderate	3.5	IIIC	9
1919	Dead	19	Male	65	Moderate	5	IIIB	9

1921	Dead	15	Male	56	Moderate	6	IIIB	9
1923	Alive	55	Male	54	Moderate	6.5	IIIB	2
1927	Dead	19	Male	67	Moderate	6	IIIB	6
1928	Alive	55	Female	52	Moderate	5.5	I	6
1929	Dead	23	Female	62	Moderate	5	IIIB	6
1990	Alive	57	Male	43	Moderate	4	IIA	9
1991	Alive	57	Male	83	Moderate	4	IIB	4
1992	Alive	56	Male	66	Moderate	4	IIA	3
1993	Dead	0	Male	82	Poor	7	IIIC	3

Table S2

Univariate and multivariate analyses of various prognosis parameters in 89 CRC patients using Cox regression model

Variable	All case	Univariate analysis		Multivariate analysis	
		HR(95% CI)	*P*	HR(95% CI)	*P*
**Gender**		0.733(0.370-1.452)	0.374		
Male	46				
Female	43				
**Age(y)**		0.952 (0.455-1.992)	0.897		
＜59	26				
≥59	63				
**Tumor size (cm in diameter)**		2.462(1.147-5.284)	**0.021**	1.788(0.829-3.859)	0.138
＜5	37				
≥5	52				
**Nodal metastasis**		3.135(1.588-6.188)	**0.001**		
N0	58				
N1/2	31				
**Differentiation**					
Well	21	2.434(1.345-4.405)	**0.003**	1.767(0.965-3.238)	0.065
Moderate	55				
Poor	13				
**AJCC Stage**		3.790(1.889-7.605)	**<0.001**	2.603(1.264-5.358)	**0.009**
I/II	56				
III/IV	33				
**CDK5 expression**		3.457(1.563-7.645)	**0.002**	2.428(1.089-5.413)	**0.030**
Low	40				
High	49				

Table S3

A list of AP-1 dependent genes related to cell proliferation and migration. (fold change>1.5, *P*<0.05)

Genes	Description	Fold change (ShCDK5/Scramble)	P-Value
FOS	Homo sapiens FBJ murine osteosarcoma viral oncogene homolog	0.50	0.001
JUN	Homo sapiens jun proto-oncogene	0.40	<0.001
VEGFA	Homo sapiens vascular endothelial growth factor A	0.47	<0.001
MMP1	Homo sapiens matrix metallopeptidase 1 (interstitial collagenase)	0.25	0.008
MYC	Homo sapiens v-myc avian myelocytomatosis viral oncogene	0.63	0.004
CD274	Homo sapiens CD274 molecule (CD274)	0.47	0.001
ZEB1	Homo sapiens zinc finger E-box binding homeobox 1	0.22	0.021
FOSL1	Homo sapiens FOS-like antigen 1	0.33	<0.001
