# Supplementary material for: CDK5 functions as a tumor promoter in human colorectal cancer via modulating the ERK5–AP-1 axis
Source: Cell Death Dis. 2016 Oct 13;7(10):e2415–. doi: 10.1038/cddis.2016.333 (PMC5133995; doi:10.1038/cddis.2016.333)
Supplement: Supplementary Figures [file cddis2016333x2.pdf]

Figure S1

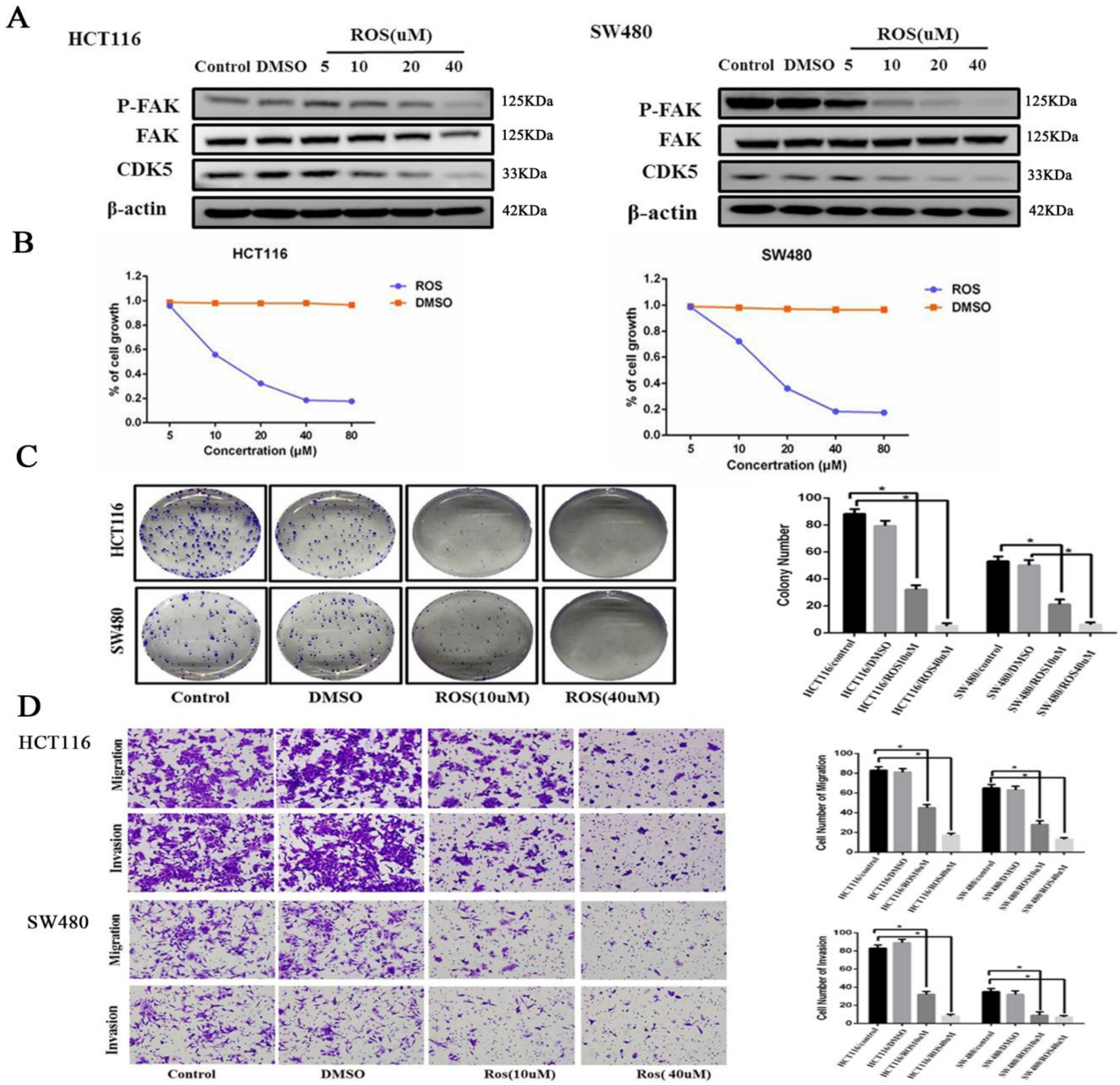

Figure S1. Treatment with CDK5 specific inhibitor roscovitine significantly inhibited CRC cells' proliferation and migration ability. (A). Expression of CDK5 and its kinase activity via detecting the protein level of p-FAK(Ser732) in HCT116 and SW480 cell lines treated with roscovitine was shown by western blotting. (B and C). CCK8 and colony formation assays were used for detecting the proliferation ability of HCT116 and SW480 by treating with a gradiently increasing conceration of roscovitine or DMSO. (D).Transwell assays were performed to detect the migration and invasion ability of HCT116 and SW480 by treating with roscovitine(10 μM, 40 μM) or DMSO. Error bars represent mean ± SD from 3 independent experiments; *, $P < 0.05$.

Figure S2

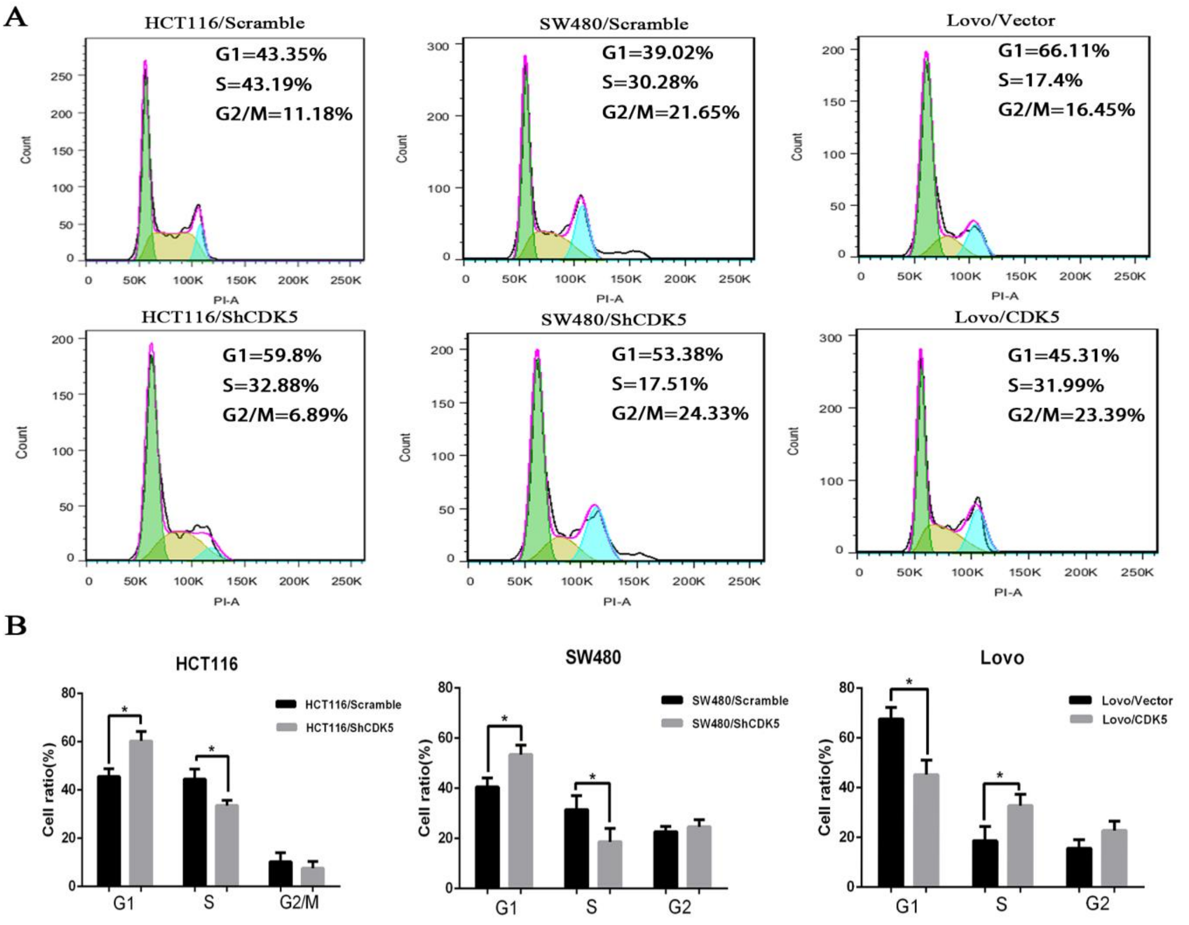

Figure S2. CDK5 accelerated cell cycle progression of CRC cells. (A) The cell cycle phases of treated cells were evaluated by flow cytometry after transfection for 48h. (B) Data was expressed as means ±SD (n=3) (* *P*<0.05).

Figure S3

A

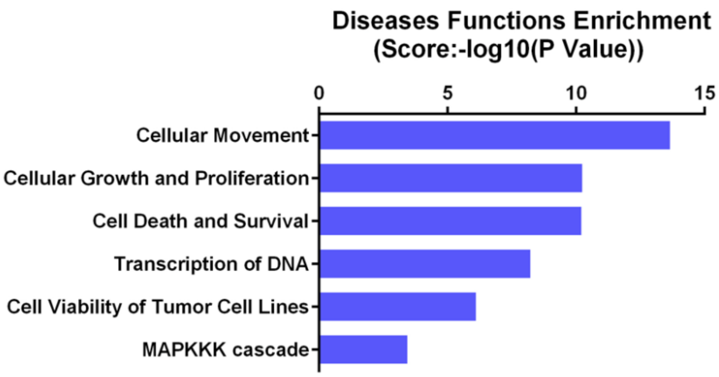

B

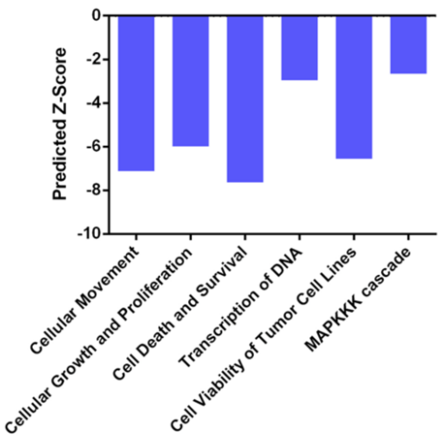

Figure S3. Knocking down CDK5 induced differential expression of genes involved in cellular proliferation, survival and movement. (A). Disease function enrichment of whole-genome expression microarray in CDK5 knocking down HCT116 cells was analyzed by Ingenuity Pathway Analysis. (B). Predicted activation state of each disease function based on the regulation z-score (Increased:The z-score is ⩾2. IPA predicts that the process or disease will increase. Decreased: The z-score ⩽-2. IPA predicts that the process or disease will decrease.) was shown.

Figure S4

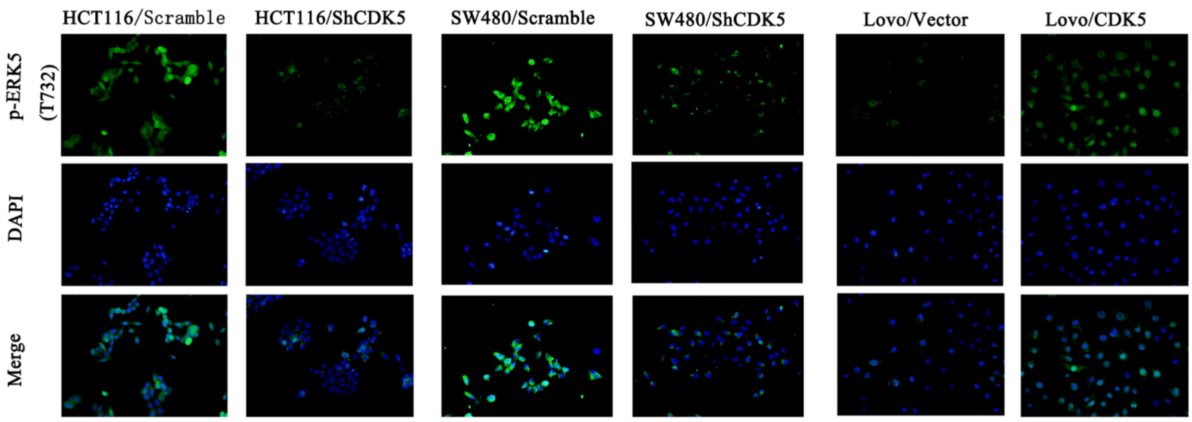

Figure S4 Immunofluorescence images of p-ERK5 (green) and DAPI (blue) in transfected CRC cell lines (×400).

Figure S5

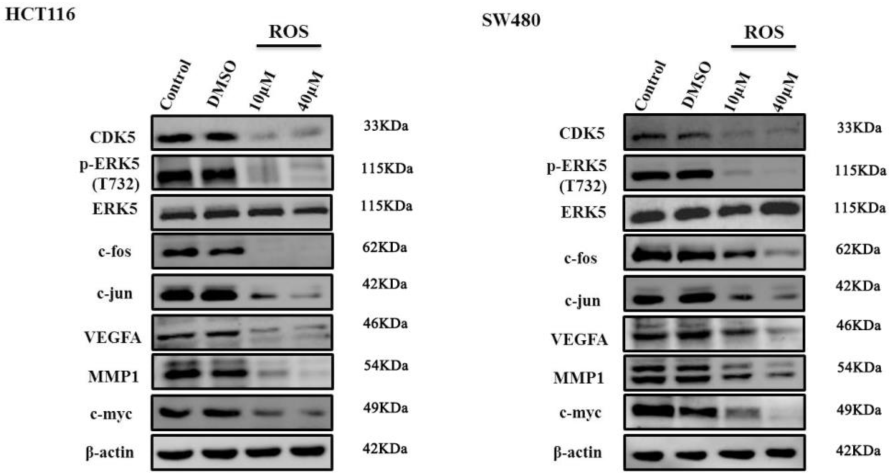

Figure S5. Whole proteins were extracted from HCT116 and SW480 treated with either roscovitine(10 μM, 40 μM) or DMSO. Western blot analysis was performed to detect the indicated protein level of the ERK5-AP-1 axis.
